# Proactive Personality as a Critical Condition for Seeking Advice and Crafting Tasks in Ambiguous Roles

**DOI:** 10.3390/bs12120481

**Published:** 2022-11-27

**Authors:** Inyong Shin, Minwoo Kim

**Affiliations:** Division of Business Administration, Pukyong National University, Busan 48513, Republic of Korea

**Keywords:** role ambiguity, proactive personality, advice-seeking, task crafting

## Abstract

In increasingly ambiguous work contexts, advice-seeking and task crafting behaviors are becoming more significant than ever before. Drawing on the uncertainty reduction theory, this study examined how role ambiguity would affect advice-seeking and task crafting. We also investigated whether a proactive personality would moderate the effects based on the capacity–willingness–opportunity model. The results, based on a two-wave design with a sample of 160 employees in South Korea, revealed that role ambiguity did not affect advice-seeking and task crafting directly. However, it was found that, as role ambiguity increased, employees with proactive personality became more involved in advice-seeking and task crafting. These findings indicate that role ambiguity serves as an opportunity for proactive employees who have the capacity and willingness to seek advice and craft tasks.

## 1. Introduction

Changes in work contexts, including flexible arrangements, autonomous conditions, and expanded empowerment, require employees to adjust to unstructured and unconventional working practices and procedures [1,2]. In the process of such adjustments, employees may feel uncertain about how to perform their work appropriately. Accordingly, they often suffer from role ambiguity, which refers to a lack of clarity in understanding the actions needed to realize proposed personal goals [3,4,5]. Ambiguous roles are particularly likely to occur in today’s complex work environment, where work responsibilities and performance are distributed among teams and team members [6].

Meanwhile, it is imperative that organizations drive innovation to survive and prosper in rapidly changing business environments. The success of innovation has been recognized as dependent on self-directed and future-focused actions in which members aim to bring about change at work [7,8,9]. In particular, in recent years, there has been a growing academic interest in the behaviors of advice-seeking or pursuing recommendations from others for a solution or process to address a challenge [10] and task crafting or changing the number, scope, and/or type of tasks performed [11]. This interest stems from evidence that such behaviors contribute to organizational innovation by enhancing creative performance [12,13].

In increasingly ambiguous work contexts, behaviors such as advice-seeking and task crafting are becoming more significant than ever before [14]. Therefore, one may ask how the ambiguous roles that employees experience at work affect the extent to which they seek advice and craft tasks. Shedding light on this inquiry helps identify whether the current situation that employees experience plays a causal role in triggering their critical actions. However, few scholars have paid attention to the effects of role ambiguity on advice-seeking and task crafting. Accordingly, this study attempts to fill this research gap by exploring how role ambiguity affects advice-seeking and task crafting.

However, it is noteworthy that ambiguity implies a weak situation in which individuals are less pressured by the environment to behave in prescribed ways and are more likely to reveal their own characteristics [14]. In other words, whether or not to take the initiative in ambiguous situations is highly likely to depend on individual dispositions [15]. Proactive personality, which refers to one’s tendency to take actions to influence changes in his or her surrounding environment [16,17], captures the dispositional components of personal initiatives [18]. Proactive personality is likely to serve as an important trait that accounts for individual differences driving work-related changes in uncertain situations. This study thus investigates whether proactive personality functions as a critical condition for seeking advice and crafting tasks in ambiguous roles.

The main purpose of this study is to examine the relationships between the ambiguous roles faced by today’s organizational members and the behaviors desired by the organizations to which they belong. In doing so, we draw on the uncertainty reduction theory [19,20] to guide our argument about the effect of role ambiguity on the likelihood that employees seek advice from their colleagues and craft their own tasks. This study also aims to identify whether proactive personality moderates the relationships of role ambiguity with advice-seeking and task crafting. We apply the capacity–willingness–opportunity model [21] to propose a rationale for the moderating effects of proactive personality in the relationships.

The following section proposes hypotheses for predicting advice-seeking and task crafting. The data collection, construct measures, and empirical results are then detailed. We finally discuss the findings of this study theoretically and practically.

## 2. Conceptual Framework of Advice-Seeking and Task Crafting

### 2.1. The Main Effect of Role Ambiguity

In general, role ambiguity, one of the role stressors, has been regarded as detrimental not only to individual well-being but also to organizational effectiveness. For example, it has been shown that employees with ambiguous roles have higher depression and turnover intention and lower job performance and organizational citizenship behavior [1,6,22,23]. Arguments regarding the negative effects of role ambiguity seem to be based on the premise that role ambiguity has a hindrance component. In other words, it is emphasized that individuals tend to evaluate ambiguous roles as threats and obstacles to work completion [24].

However, it has been overlooked that ambiguity can provide an individual with the driving force to lower it. According to the uncertainty reduction theory, uncertainty causes individuals to exhibit certain behaviors to reduce it [19,20]. In other words, this theory underscores the notion that uncertainty reduction can be one of the main drivers for some behaviors. In this regard, some scholars insist that when employees experience uncertain situations, they are likely to attempt to exhibit self-directed and future-focused actions for change at work with the aim of reducing uncertainty [14]. These attempts are expressed as actively challenging rather than passively adapting to unfavorable situations. Accordingly, workers in unclear and equivocal situations tend to clarify the purpose and meaning of what they do and influence their work contexts in order to resolve uncertainty [25,26]. In the same vein, employees with ambiguous roles are expected to adopt active and voluntary approaches which are characterized by behaviors such as understanding work environments to make appropriate judgments, improving given work methods, and developing potential work demands [27,28]. Therefore, in ambiguous roles, employees are likely to pursue advice from their colleagues to improve the quality of their decision-making and redesign their tasks to create personally optimized work environments.

Taken together, we expect ambiguous roles to provide employees with opportunities to seek advice from their colleagues and to craft their own tasks. Although there is no substantial evidence that role ambiguity influences advice-seeking and task crafting, previous research has shown that role ambiguity has positive correlations with behaviors related to obtaining information and altering tasks [29,30]. Accordingly, we suggest the following hypothesis:

**Hypothesis** **1.** *Role ambiguity will have positive effects on (a) advice-seeking and (b) task crafting.*

### 2.2. The Moderating Effect of Proactive Personality

It has been generally thought that an individual’s behavior or performance is determined by the interplay of his or her capacity, willingness, and opportunity [21]. We anticipate that these three basic dimensions of behavior, also called the capacity–willingness–opportunity model, can be applied to accounting for advice-seeking and task crafting behaviors. Specifically, we posit that proactive employees are more likely to engage in pursuing advice from their colleagues and redesigning their own tasks by leveraging their capacity and willingness and taking advantage of ambiguous roles as opportunities.

Proactive personality is an innate characteristic of an individual and is one of the main factors explaining his or her thinking and behavior [16]. In particular, proactive employees are known to have a greater sense of work-role self-efficacy [31]. Workers who are highly proactive tend to take control of difficult situations and demonstrate action-oriented attitudes toward their work [32], which contributes to improving their capacity to drive change [27]. In addition, proactive personality plays a pivotal role in inducing autonomous motivation [33], which is a significant driver in generating inventive efforts and novel approaches [34]. Therefore, proactive employees are willing to adopt future- or problem-oriented coping ways [27]. In sum, proactive employees are capable of and willing to develop ways to find solutions, such as seeking advice from their colleagues and redesigning their own tasks.

Meanwhile, while non-proactive individuals tend to be passive and prefer to adapt to situations rather than change them, proactive individuals tend to identify and seize opportunities to change things [35]. That is, proactive personality serves as a precursor to discovering positive opportunities from situational cues and shaping work environments that are consistent with individual needs [16,31]. Therefore, proactive employees are more likely to recognize role ambiguity as an opportunity to make differences and demonstrate novel patterns of behaviors in the workplace, rather than as one of the stressors that evoke negative emotions and cause them to stick to existing behaviors.

As discussed above, role ambiguity is likely to create an opportunity to seek advice and craft tasks. However, not all employees are expected to respond to the opportunity in the same way. Instead, we believe that proactive employees are capable of translating and willing to translate role ambiguity into advice-seeking and task crafting. In other words, employees who utilize their capacity and willingness and take ambiguous roles as opportunities based on their proactive propensity are more likely to seek relevant information and advice from their peers and alter the type and scope of their tasks. Therefore, we suggest the following hypothesis:

**Hypothesis** **2.** *Proactive personality will moderate the positive effects of role ambiguity on (a) advice-seeking and (b) task crafting, such that as role ambiguity increases, employees with a proactive personality are more likely to seek advice from their colleagues and craft their tasks.*

**Figure 1:**
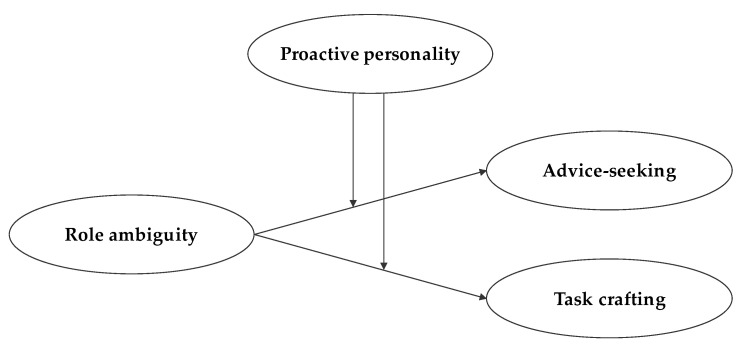
Conceptual model.

Figure 1 depicts the conceptual model of this study graphically. The model shows that the likelihood of advice-seeking and task crafting will be determined by role ambiguity and strengthened by proactive personality.

## 3. Method

### 3.1. Sample and Procedure

Role ambiguity is evident in all job positions regardless of organizational changes, and behaviors such as advice-seeking and task crafting are commonly observed in workplaces [1,14]. We thus decided to collect data from employees at several small and medium-sized companies across various industries in South Korea. To clarify the effects of role ambiguity on advice-seeking and job crafting, a two-wave design in which data were collected with a six-month interval was adopted. The back-translation procedure [36] was utilized to confirm that the measurement items for each variable on the questionnaires were identical to the original. Prior to the first survey, we met with the management and human resource management staff of each company to explain the survey’s purpose and contents. After the employees of each company had given consent, they completed the survey. The same procedure was followed in the second survey.

While 239 participants completed the first survey (Time 1), 194 responded to the second survey six months later (Time 2). The hypotheses were tested with a final sample of 160 participants who had responded to all measures in both the first and second surveys. The demographic characteristics of the final sample were as follows: The participants’ mean age was 40.30 years, 61.88% were male, 38.13% had a bachelor’s degree, and they had worked in their current organization for an average of 6.08 years.

### 3.2. Measures

The measures were taken at two different time points to reduce the possible impact of common method variance by separating independent and moderating variables from dependent variables [37]. At Time 1, role ambiguity, proactive personality, and demographics were measured. At Time 2, advice-seeking and task crafting were measured. A five-point scale, ranging from 1 (strongly disagree) to 5 (strongly agree), was employed to measure all the main variables with the exception of advice-seeking.

#### 3.2.1. Role Ambiguity

A three-item scale adapted from Bowling et al. [38] was used to measure role ambiguity. A sample item is “The requirements of my job are not always clear.” The Cronbach’s alpha was 0.78.

#### 3.2.2. Proactive Personality

The six-item version of the Proactive Personality Scale, employed in previous studies [39,40], was used to evaluate proactive personality. A sample item is ‘‘No matter what the odds, if I believe in something I will make it happen”. The Cronbach’s alpha was 0.84.

#### 3.2.3. Advice-Seeking

A social network survey was employed to measure the extent to which employees sought advice. The questionnaire was based on the roster method and included the names of all the employees in the organization for which they worked [41,42]. Compared to the name generator method, which identifies the alters mentioned by the focal individual, the roster method, which includes all actors in the organization, is less likely to be distorted by perceptual biases [43]. Accordingly, the roster method is recognized as accurate and reliable by improving employee recall [41]. The respondents were required to look down the list of organizational members and indicate how often they had sought work-related information and advice from each member of their organization during the previous six months [44,45] by selecting one of the following three categories: 0 (never), 1 (little), 2 (much). An advisory relation was noted if they selected “little” or “much”. Following the previous study [46], outdegree centrality was calculated by employing UCINET 6 to determine each participant’s advice-seeking value. It was then normalized to reflect the number of different members in each organization.

#### 3.2.4. Task Crafting

Task crafting was assessed using Slemp and Vella-Brodrick’s [47] six-item scale. An example item is “I introduce new approaches on my own to improve my existing work”. The Cronbach’s alpha was 0.92.

#### 3.2.5. Control Variables

The participants were recruited from different organizations in various industries. Accordingly, the industry to which each organization belonged (0 = manufacturing, 1 = service) and its size (the number of organizational members) were controlled. Individual demographic variables that may have affected employees’ advice-seeking and task crafting were also included as controls [12,46]. Specifically, we controlled for age (in years), gender (0 = female, 1 = male), educational level (1 = high school, 2 = two-year college, 3 = four-year university, 4 = master, 5 = doctor), and organizational tenure (in years).

## 4. Results

### 4.1. Reliability and Validity Testing

Cronbach’s alpha was employed to identify the reliability of the variables. The alpha values ranged from 0.78 to 0.92, indicating that they had satisfactory internal consistency [48]. To assess the convergent and discriminant validity of the variables, we conducted a confirmatory factor analysis using AMOS 21. The model showed an acceptable fit (χ^2^_(86)_ = 164.61, CFI [comparative fit index] = 0.94; TLI [Tucker–Lewis index] = 0.93; RMSEA [root mean square error of approximation] = 0.08; SRMR [standardized root mean square residual] = 0.06). The standardized factor loading value of each item ranged from 0.51 to 0.90, and the composite reliability of all the variables ranged from 0.83 to 0.95. The average variance extracted values of each variable, which ranged from 0.59 to 0.76, were larger than the squared values of the correlation coefficients between the variables. It was thus judged that the variables included in this study had acceptable levels of convergent and discriminant validity [49,50].

### 4.2. Hypothesis Testing

The means, standard deviations, and correlations for all variables are presented in Table 1. To test the hypotheses, we conducted a three-step hierarchical multiple regression analysis using SPSS 27. First, the control variables (i.e., industry, organizational size, age, gender, educational level, and organizational tenure) were entered. Subsequently, the independent variable (i.e., role ambiguity) and the moderating variable (i.e., proactive personality) were entered. Finally, the interaction term created by multiplying the mean-centered role ambiguity and proactive personality was entered.

As shown in Table 2, role ambiguity was positively but not significantly related to advice-seeking (Model 2, *β* = 0.10, n.s.) and task crafting (Model 5, *β* = 0.07, n.s.), indicating that role ambiguity does not affect advice-seeking and task crafting directly. Therefore, hypothesis 1a,b were not supported.

**Figure 2:**
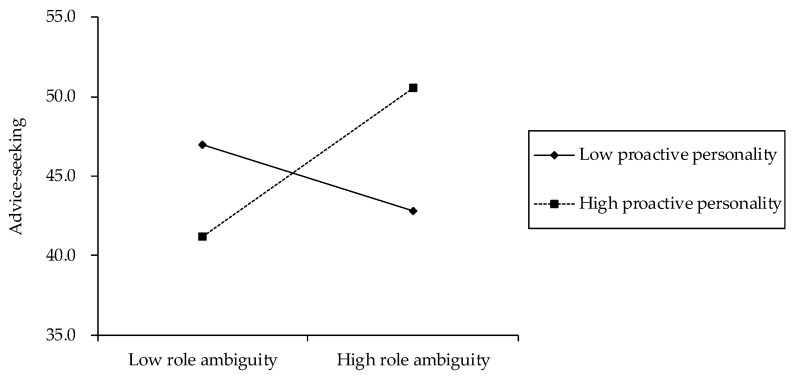
The moderating effect of proactive personality on the relationship between role ambiguity and advice-seeking.

**Figure 3:**
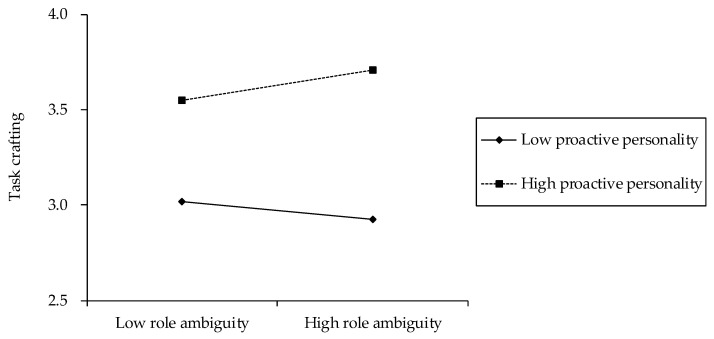
The moderating effect of proactive personality on the relationship between role ambiguity and task crafting.

The coefficients of the interaction term between role ambiguity and proactive personality were significantly positively related to advice-seeking (Model 3, *β* = 0.16, *p* < 0.05) and task crafting (Model 6, *β* = 0.13, *p* < 0.05). Therefore, the results supported hypothesis 2a,b, in that proactive personality would moderate the effects of role ambiguity on advice-seeking and task crafting. To investigate the nature of such moderation effects, the significant interactions were plotted for low and high levels of proactive personality, that is, one standard deviation below and above its mean. As depicted in Figure 2, while the relationship between role ambiguity and advice-seeking was positive for high proactive personality, the relationship was negative for low proactive personality. The slope test further revealed that the positive slope for high proactive personality was significant (*b* = 6.00, *p* < 0.05), but the negative slope for low proactive personality was not significant (*b* = −2.71, n.s.). The interaction plots in Figure 3 indicated that the relationship between role ambiguity and task crafting was positive for high proactive personality and negative for low proactive personality. Furthermore, although the positive slope for high proactive personality was significant (*b* = 0.10, *p* < 0.05), the negative slope for low proactive personality was not significant (*b* = −0.06, n.s.); these results confirmed hypothesis 2a,b.

## 5. Discussion

In this study, we examined how role ambiguity that employees experience at work would influence their behaviors of seeking advice from their colleagues and crafting their own tasks as well as under what conditions such influences appear. Two-wave data from 160 employees in South Korea were employed to test the hypotheses. The results revealed that role ambiguity did not have any direct effects on advice-seeking and task crafting. However, it was shown that proactive personality moderated the effects of role ambiguity on advice-seeking and task crafting. Therefore, these results indicate that role ambiguity enables proactive employees, but not all employees, to engage in advice-seeking and task crafting behaviors.

### 5.1. Theoretical Contributions

Changes in business environments require employees to demonstrate self-directed and future-focused behaviors conducive to organizational innovation. In particular, behaviors such as seeking advice and crafting tasks are acknowledged as contributing to organizational innovation by enhancing individual creativity [12,13]. Accordingly, there is growing scholarly interest in what factors promote advice-seeking and task crafting. Previous research has shown that individual characteristics, including gender, anxiety, and hierarchical rank, as well as organizational characteristics such as acquisition, serve as antecedents of advice-seeking [51,52,53,54]. In addition, it has been found that growth needs and creative self-efficacy as individual characteristics, job autonomy as job characteristics, and organizational support and human resource management systems as organizational characteristics positively influence task crafting [55,56,57,58]. However, prior studies have neglected to investigate the phenomenon that organizational members are exposed to ambiguous roles due to changes in work context and the effects of these ambiguous roles on advice-seeking and task crafting. Therefore, this study attempted to fill this research gap by disambiguating the potential roles of role ambiguity in predicting advice-seeking and task crafting. This study is theoretically meaningful in that it sought to examine whether the situation experienced by organizational members would lead to the behaviors desired by organizations based on uncertainty reduction theory.

According to the analyzed results, the relationships of role ambiguity with advice-seeking and task crafting were positive; however, the relationships were not significant. These results may be because the strain from ambiguous roles induces routinization. Employees with uncertain role expectations are vulnerable to strain symptoms, including anxiety, anger, and depression [4,59]. When employees suffer work-related strain, they tend to employ routinized behavioral patterns by adopting passive coping styles in an attempt to manage such strain [60]. The more they become immersed in the behavioral patterns, the less involved they become in the challenging aspects of their work [61]. Consequently, it is inferred that the effects of role ambiguity on advice-seeking and task crafting are offset because role ambiguity may induce uncertainty reduction but may also lead to routinization.

Scholars have emphasized that it is crucial to investigate how individual and situational factors interact to stimulate self-directed and future-focused behaviors aimed at making changes in the workplace in order to fully understand the behaviors [14,17]. With the need for such research in mind, we revealed that role ambiguity as a situational antecedent plays a pivotal role in predicting advice-seeking and task crafting when combined with proactive personality as a dispositional condition. In this regard, by applying the capacity–willingness–opportunity model, we suggested a rationale for employees with proactive personality to pursue advice and redesign tasks in ambiguous roles. Consequently, the findings of this study imply that ambiguous roles act as opportunities for proactive employees to tap into their capacity and willingness to engage in advice-seeking and task crafting behaviors. This study contributes to the expansion of the role ambiguity and proactive personality literature by establishing a comprehensive linkage between role ambiguity, proactive personality, advice-seeking, and task crafting. Our finding that proactive employees seek more advice and craft more tasks in ambiguous roles is consistent with the argument that role ambiguity may provide room for individuals to decide what to do and that proactive personality can play a stronger role in unstructured environments [62].

### 5.2. Practical Implications

The results of this study offer some practical implications for managers and human resource management staff in contemporary organizations. First, it is noteworthy that, although roles in the workplace have generally become unclear, each employee perceives role ambiguity differently. Therefore, it is desirable for organizations to monitor their members’ levels of role ambiguity. It can be helpful to conduct regular interviews with managers and subordinates, as well as employee opinion surveys. Furthermore, it can be necessary to manage and develop tolerance and familiarity with ambiguous roles. For example, by placing employees on risk-free, unstructured committees or project teams, they can become accustomed to ambiguous situations and gradually learn to embrace ambiguity as they gain work experience [2]. Second, it is advisable to employ or place proactive employees in jobs in which roles may be ambiguous. In the case of new employment, department placement, and project team launch, it may be beneficial to identify applicants’ proactive personality and utilize the results for selection or placement. By delegating highly ambiguous work-related roles to proactive employees, it will be possible to allow them to come up with solutions on their own. These approaches will contribute to promoting organizational change and innovation by facilitating their members’ change-directed behaviors.

### 5.3. Limitations and Future Research Directions

The model of this study needs further theoretical elaboration and subsequent testing. Specifically, a sample of 160 employees working in Korean companies was used to test the hypothesis of this study. In order to increase the statistical confidence and generalizability of the results of this study, it is necessary to collect and analyze more data from employees of various nationalities. In addition, it is necessary to expand the scope to other personality factors other than the proactive personality construct, which was the focus of this study. For example, under conditions of ambiguity, neurotic employees may plan and spend additional effort to achieve desired outcomes, and open employees may recognize and embrace a wider range of possibilities. Therefore, neuroticism and openness to experience are likely to moderate the effects of role ambiguity on advice-seeking and task crafting [14]. We hope that further studies on these issues will be performed. Finally, while advice-seeking and task crafting have been examined in this study as specific forms of self-directed and future-focused behaviors aimed at making a difference at work, previous studies have shown that the ways in which employees express such behaviors in the workplace vary widely. Accordingly, further research is recommended to examine how ambiguous roles influence different types of proactive behaviors, including taking charge, expressing voice, and building social networks [63,64,65].

## 6. Conclusions

This study implies that the transition of role ambiguity into work-related change behaviors such as advice-seeking and task crafting is not an automatic process. Instead, it depends on an individual unique trait, proactive personality, to take the uncertainty that arises from increasing role ambiguity as an opportunity and elicit the capacity and willingness to engage in such behaviors.

## Figures and Tables

**Table 1:** Means, standard deviations, and correlations.

Variables	Mean	SD	1	2	3	4	5	6	7	8	9
1. Industry	0.71	0.46									
2. Organizational size	23.26	5.73	−0.35 **								
3. Age	40.30	9.91	−0.24 **	0.05							
4. Gender	0.62	0.49	0.00	0.18 *	0.10						
5. Educational level	2.21	0.99	0.25 **	−0.40 **	−0.12	−0.06					
6. Organizational tenure	6.08	5.84	0.11	0.29 **	0.32 **	−0.03	−0.15				
7. Role ambiguity	2.52	0.78	−0.17 *	0.05	0.02	−0.08	−0.12	−0.11			
8. Proactive personality	3.37	0.58	0.21 **	−0.15	0.05 *	0.16 *	0.15	0.16 *	−0.16 *		
9. Advice-seeking	45.37	27.22	0.34 **	−0.24 **	−0.13 *	0.15	0.24 **	−0.04	0.01	0.16 *	
10. Task crafting	3.30	0.62	0.24 **	−0.20 *	0.03 *	0.15	0.31 **	0.20 *	−0.08	0.64 **	0.17 *

Note. *N* = 160. All tests are two-tailed, * *p* < 0.05, ** *p* < 0.01.

**Table 2:** Regression results on advice-seeking and task crafting.

Variables	Advice-Seeking			Task Crafting		
	Model 1	Model 2	Model 3	Model 4	Model 5	Model 6
Industry	0.24 **	0.25 **	0.26 **	0.06	0.01	0.02
Organizational size	−0.14	−0.13	−0.13	−0.20 *	−0.10	−0.10
Age	−0.08	−0.09	−0.08	−0.03	−0.04	−0.03
Gender	0.19 *	0.19 *	0.18 *	0.22 **	0.11	0.10
Educational level	0.13	0.14	0.14	0.27 **	0.22 **	0.23 **
Organizational tenure	0.03	0.03	0.03	0.31 **	0.20 **	0.19 **
Role ambiguity		0.10	0.05		0.07	0.03
Proactive personality		0.05	0.02		0.56 **	0.53 **
Role ambiguity × Proactive personality			0.16 *			0.13 *
R^2^	0.18	0.19	0.21	0.23	0.50	0.51
F	5.72 **	4.54 **	4.55 **	7.70 **	18.71 **	17.48 **

Note. *N* = 160. All tests are two-tailed, * *p* < 0.05, ** *p* < 0.01.

## Data Availability

The data presented in this study are available upon request from the corresponding author.
